# Supplementary material for: Identification of Freezing-Responsive microRNAs and Their Targets in Chinese Jujube by Small RNA and Degradome Sequencing
Source: Int J Mol Sci. 2026 Sep 4;27(17):7912. doi: 10.3390/ijms27177912 (PMC13566890; doi:10.3390/ijms27177912)
Supplement: Supplementary file 1 [file ijms-27-07912-s001.zip › Supplementary Material S2/novel_miR_33.pdf]

Provisional ID : novel_miR_33
Score total : 30
Score for star read(s) : 3.9
Score for read counts : 27.7
Score for mfe : 0.6
Score for randfold : -2.2
Score for cons. seed :
Total read count : 66
Mature read count : 54
Loop read count : 10
Star read count : 2

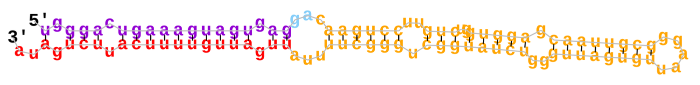

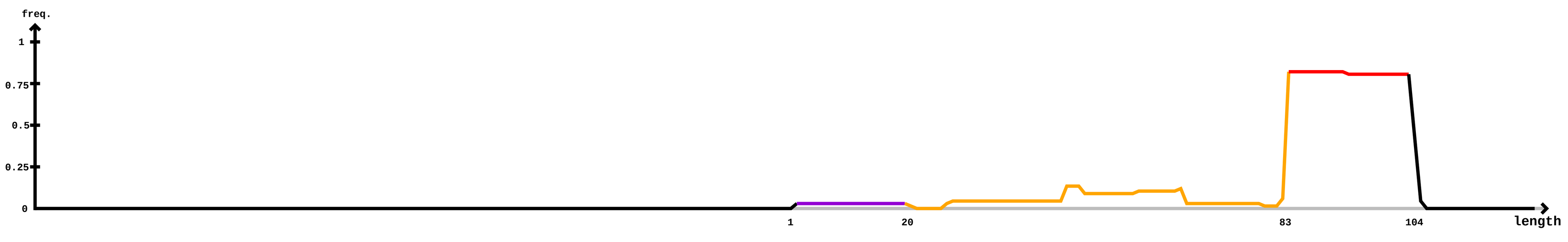

Star Mature

```
5'- gcgcuucagaaauucaacguuucguauaaucgauuaaacgggucgaucccaucgaagcuaucgagguuugaagcaagcgcuuucgaagguaauucacuuugugggaagcccuugauugugugcaaugggacugaaaguagugaggacaaguccuugucugguggagcaauugcgggaauugugauugggucuauggcugggcuuuuauugauuguuuucauucugauauuucuauguaagaaaagaaaga -3' obs
    gcgcuucagaaauucaacguuucguauaaucgauuaaacgggucgaucccaucgaagcuaucgagguuugaagcaagcgcuuucgaagguaauucacuuugugggaagcccuugauugugugcaaugggacugaaaguagugaggacaaguccuugucugguggagcaauugcgggaauugugauugggucuauggcugggcuuuuauugauuguuuucauucugauauuucuauguaagaaaagaaaga     exp
    ..((((((((..(((...((((((....(((((((.....))))))).....))))))....))).))))))))..((((..((((.(((..(((((...)))))..))).))))..))))...((.(((.((((((((((.((...((((((..(((..(((((.((((((((.....))))))))..)))))))).))))))...)).)))))))))).))).)).(((((.....)))))....... reads mm sample
    .............................................................................................................................ugggacugaaaguagugag.......................................................................................................... 2 0 JCK-2
    ......................................................................................................................................................uccuugucugguggagcaauugc............................................................................. 2 0 LT-2
    .......................................................................................................................................................ccuugucugguggagcaauugc............................................................................. 1 0 LT-1
    ..........................................................................................................................................................................ugcgggaauugugauugggu............................................................ 6 0 LT-2
    ......................................................................................................................................................................................gauugggucuauggcugggcu............................................... 1 0 LT-3
    .............................................................................................................................................................................................ucuauggcugggcuuuuauugauuguuu................................. 1 0 LCK-3
    ..............................................................................................................................................................................................................auugauuguuuucauucugauauu.................... 1 0 JCK-3
    ..............................................................................................................................................................................................................auugauuguuuucauucugauauu.................... 2 0 JCK-2
    ...............................................................................................................................................................................................................uugauuguuuucauucugaua...................... 29 0 LCK-2
    ...............................................................................................................................................................................................................uugauuguuuucauucugaua...................... 22 0 LCK-1
```
